# Gender disparities and associated factors to intention to getting a second dose of COVID-19 AstraZeneca vaccine among adult populations in selected facilities of Lusaka, Zambia

**DOI:** 10.1371/journal.pgph.0000265

**Published:** 2022-07-20

**Authors:** Choolwe Jacobs, Nedah Chikonde Musonda, Deborah Tembo, Miyanda Simwaka, Evelyn Mwamba, Sody Mweetwa Munsaka, Samson Shumba

**Affiliations:** 1 Department of Epidemiology and Biostatistics, School of Public Health, University of Zambia, Lusaka, Zambia; 2 Zambia National Public Health Institute, Lusaka, Zambia; 3 The University Teaching Hospital, Lusaka, Zambia; 4 Department of Biomedical Sciences, School of Health Sciences, University of Zambia, Lusaka, Zambia; Bahauddin Zakariya University, PAKISTAN

## Abstract

As COVID-19 vaccines are becoming more available, there is also a growing need to understand the population receiving the doses, existing inequalities and the intention to getting the second vaccine dose among the populations that receive the vaccines. We evaluated gender inequalities and intention to uptake of the second dose of COVID-19 AstraZeneca vaccine among adult populations in selected urban facilities of Lusaka, Zambia. A cross-sectional study design was conducted between May and June 2021 among adults who received AstraZeneca vaccine from three selected urban facilities of Lusaka, Zambia. Phone-based interviews were conducted 6 weeks after the first dose of the vaccine. Descriptive analysis and mixed-effect logistic regression were done using STATA version 16.2. Of the 1321 adults who had received AstraZeneca vaccine, 868 respondents completed the questionnaire. About, 47% (408/868) were females and 53% (460/868) were males. Median age in the study was 40 years. Majority of males were educated (54%) and employed (57%). Furthermore, majority of females that got the first dose of AstraZeneca reported experiencing side effects (76.98%) compared to males (64.24%). Among study participants, 93.7% intended to receive the AstraZeneca vaccine, of whom 46.7% (380/814) were females and 52.9% (434/814) were males. Majority of participants that did not intend to get a second dose were not married (55.56%). Only age (AOR, 1.05; 95% CI, 1.02–1.08) predicted intention to getting a second dose of AstraZeneca vaccine. We found important gender-dependent differences in the side effects reported by females that received the first dose of Astra Zeneca. Finding that intention to get the second dose of the vaccine increased with age suggests a need for enhancing COVID-19 vaccination programmes targeting young people and a need for further research to identify specific adverse effects of COVID-19 Astra Zeneca vaccines.

## Introduction

Coronavirus disease 2019 (COVID-19) pandemic caused by the novel severe acute respiratory syndrome coronavirus 2 (SARS-COV-2) [1], has continued to ravage the world impacting global health and the global economy across all populations including Zambia [2, 3]. With the pandemic spreading wide and fast across the world, it prompted the scientific community to find vaccines, that would be the most effective way of controlling the pandemic [4]. Vaccinations will be extremely vital in controlling the COVID-19 pandemic. To achieve ‘herd immunity, approximately 67% to 80% needs to be achieved to reduce the spread of the disease [5]. One of the first vaccines introduced in most of the developing countries including Zambia is the Oxford-AstraZeneca vaccine which is administered through two doses [6, 7]. The vaccine is said to not function well if an individual missed out on the second dose or delayed as prescribed [8]. However, the success of the vaccination programmes depends on public acceptance of the vaccines [9].

As COVID-19 vaccines are now becoming more available, one of the interests has been understanding the characteristics of the population receiving the doses, the inequalities, and the intentions to getting the second vaccine among the populations that receive the vaccine. Despite the importance of the vaccines as a control measure, there has been a growing concern of vaccine hesitancy defined as a reluctance or refusal to be vaccinated [10]. A study conducted among undergradue students revealed a lower acceptance rate of 24.5%. With the rapid development and launch of the vaccines, the concerns of acceptance and gender disparities remains critical issues [15].

Inequalities and hesitancy in the uptake of vaccines are a challenge in achieving herd immunity as desired by WHO. Despite being inconsistent, previous studies have reported gendered differences in vaccine uptake [11]. A systematic review showed that men were more likely to get Covid-19 vaccine compared to women [12].

Gender inequality has become a critical part of COVID-19 vaccination. Findings in a study conducted across 16 countries show that women were not only less likely to be vaccinated but also likely to feel vaccines are indeed safe. In developing countries, women are less likely to get COVID-19 vaccines than men [13], due to household chores and socio economic factors that limit access to the health facilities such as lack of transportation [14, 15]. In Iraq for instance, 55% of the women compared to 42% of men reported to travel more than 30 minutes to get a vaccine. Meanwhile, in Malawi, women are 4 times less likely to trust a vaccine compared to men due to fears regarding fertility and similar statistics around the world [16].

In response to the rampant pandemic, Zambia started its vaccine administration with Astra Zeneca in April 2021. Evidence on gender disparities in the population that received the first round of Astra Zeneca vaccine, including their intention to receive the second round of the vaccine is limited. The purpose of this study was to evaluate gender inequalities and intention to uptake of the second dose of COVID-19 Oxford-AstraZeneca vaccine among the adult populations of Lusaka, Zambia.

## Methods

### Study design, area, and period

A cross-sectional study was conducted among adults who received the first dose of AstraZeneca vaccine in May 2021, and were due for the second dose in 8 weeks from the day they got the first one. Interviews were conducted between the 15^th^ and 22^nd^ of June 2021. Participants were recruited from three sites including the University Teaching Hospital (UTH), Chilenje Level One Hospital, and Chelstone Level One Hospital. These sites are among the health facilities in Lusaka with large catchment populations.

### Study population, sample size determination and sampling procedure

To achieve a minimum power of 80%, the study used a proportion of 38% vaccine hesitancy [17], a design effect of 1.5 and non response set at 10% to give a minimum required sample size of 603. The required minimum sample size was set at 400 from UTH and 100 each from Chilenje and Chelstone Clinic based on the catchment population in each health facility. However, all the 1321 adults, both males and females, 18 years old and above, and with documented contact details who got the first dose of Oxford-AstraZeneca Vaccine from the three health facilities between the 4^th^ and 7^th^ of May 2021 were eligible and enrolled in the study.

### Study variables and data collection

The outcome variable in the study was intention to getting a second dose of Oxford-AstraZeneca vaccine dose. Social demographic and clinical factors were collected as independent variables.

Data was collected through telephone interviews using a structured questionnaire on google forms which was synchronized with the google sheet. Data from google sheet was downloaded to microsoft excel sheet where data was cleaned and uploaded to Stata version 16 for analysis. A pilot study was conducted to validate the questionnaire. Nine trained research assistants participated in data collection. Both English and local languages were used in conducting interviews.

### Data analysis

Descriptive statistics were done which included normality test using Shapiro Wilk test and a Wilcoxon rank-sum test was used to determine the association between age and categorical variables. A Chi-square or a Fisher’s exact test was used to measure associations between categorical variables. To determine the predictors of intention to receive the second dose of AstraZeneca COVID-19 vaccine a mixed effect logistic regression was used. This was to take into account the variations between clusters (the health facilities). Akaike Information Criterion (AIC) and Bayesian Information Criterion (BIC) were computed together with the likelihood ratio test to come up with the best-fit model. All tests in the study were conducted at 5% level of significance.

## Results

### Characteristics of the sample

Overall, 868 participants of the 1321 eligible adults that received the AstraZeneca COVID-19 vaccine from 3 facilities of Lusaka, answered telephone calls and completed the questionnaire. The distributions of participants from the three facilities was as follows; Chelstone Zonal Hospital 19.35% (168/868), Chilenje General Hospital 14.99% (130/868), and UTH 65.67% (570/868).

### Characteristics of participants by sex

Findings in the study show that 52.88% (459/863) of males got the first dose of AstraZeneca vaccine compared to 47.12% (409/863) females. The median age for males (40; IQR, 31–49) and females (40; IQR, 30–51) that got the first dose of Astra Zeneca vaccine was not different. Majority of males that received the vaccine were not married (69.50%), while among females there was no difference by marriage.

Furthermore, a majority of the males (80.39%) in the study were employed compared to females (68.72%). They were also significant gender differences (p<0.0) in the proportion of participants reporting side effects and having pre-existing medical conditions. As shown on Table 1, 76.98% (311/409) of females reported side effects after getting the first dose of Astra Zeneca compared to 64.24% (291/453) of males.

**Table 1:** Background characteristics of participants by sex.

Characteristics	Sex		P-value
	Females (N = 409)	Males (N = 459)	
**Age (Median, IQR) years**	40 (30 – 51)	40 (31 – 49)	0.861^r^
	**n (%)**	**n (%)**	
**Marital Status**			
Unmarried	205 (50.25)	140 (30.50)	**<0.0001** ^**c**^
Married	203 (49.75)	319 (69.50)	
**Education**			
Upto Primary	15 (3.71)	11 (2.43)	0.194^c^
Secondary	83 (20.54)	77 (17.00)	
Higher	306 (75.74)	365 (80.57)	
**Occupation**			
Unemployment	127 (31.28)	90 (19.61)	**<0.0001** ^**c**^
Employed	279 (68.72)	369 (80.39)	
**Race**			
Non Black	27 (6.62)	48 (10.53)	**0.027** ^**c**^
Black	381 (93.38)	408 (88.89)	
**Alcohol**			
No	247 (60.69)	220 (48.25)	**<0.0001** ^**c**^
Yes	160 (39.31)	236 (51.75)	
**Took a COVID Test**			
No	153 (37.41)	192 (41.83)	0.184^c^
Yes	256 (62.59)	267 (58.17)	
**Pre-existing medical condition**			
No	279 (68.22)	350 (76.25)	**0.008** ^**c**^
Yes	130 (31.78)	109 (23.75)	
**Currently on medical Treatment**			
No	237 (66.39)	293 (74.74)	**0.012** ^**c**^
Yes	120 (33.61)	99 (25.25)	
**Side Effects**			
No	93 (23.02)	162 (35.76)	**<0.0001** ^**c**^
Yes	311 (76.98)	291 (64.24)	

r = Ranksum

c = Chi square

f = Fishers’ exact

### Characteristics of participants and intention to getting a second dose of AstraZeneca vaccine

The study investigated the associations of socio-demographic and clinical characteristics of participants with intention to getting a second dose of AstraZeneca vaccine. We observed a median age of 30 (IQR, 24–38) years for those who did not intend on getting the second dose compared to 40 (IQR, 31–51) years for those who had clear intentions of getting the second dose of vaccine. Among the participants that intended to get the second dose of the vaccine, majority were males 52.9% (434/814) compared to females 46.7% (380/814). However, the difference between the two was not statistically different. See Table 2.

**Table 2:** Characteristics of participants and intention to getting a second dose of AstraZeneca vaccine.

Variables	Intention to getting a second dose		P-value
	No (N = 54)	Yes (N = 814)	
**Age; Median (IQR) years**	30 (24 – 38)	40 (31 – 51)	**<0.0001** ^**r**^
	**n (%)**	**n (%)**	
**Sex**			
Females	29 (53.70)	380 (46.68)	0.317^c^
Males	25 (46.30)	434 (52.88)	
**Marital Status**			
Not married	30 (55.56)	315 (38.75)	**0.015** ^**c**^
Married	24 (44.44)	498 (61.25)	
**Race**			
Non Black	3 (5.56)	72 (8.89)	0.615^f^
Black	51 (94.44)	738 (91.11)	
**Education**			
Upto Primary	2 (3.70)	24 (2.99)	0.163^c^
Secondary	15 (27.78)	145 (18.06)	
Higher	37 (68.52)	634 (78.95)	
**Occupation**			
Unemployment	17 (31.48)	200 (24.66)	0.603^c^
Employed	37 (68.52)	611 (75.34)	
**Pre-existing medical Condition**			
No	44 (81.48)	585 (71.87)	0.126^c^
Yes	10 (18.52)	229 (28.13)	
**Currently on medical treatment**			
No	32 (78.05)	498 (70.34)	0.291^c^
Yes	9 (21.95)	210 (29.66)	
**Side Effects**			
No	32 (78.05)	498 (70.34)	0.291^c^
Yes	9 (21.95)	210 (29.66)	

r = Ranksum

c = Chi square

f = Fishers’ exact

Furthermore, majority of participants (78.95%) who had intentions of getting a second dose of the vaccine had a higher level of education, similar to those who had no intentions of getting the second dose (68.52%). Notably, the majority of participants who did not intend to get a second dose of the vaccine were not married (55.56%). As shown on Table 2, the current study also found that only age and marital status led to a statistically significant difference (P<0.05) in intention to get a second vaccine. While there was no statistically significant difference in intention to get a second dose of Astra Zeneca dose by gender, race, education, occupation, pre-existing medical condition, being on treatment and side effects (P>0.05).

### Multivariable mixed effect logistic regression model

The study explored the mixed-effect logistic regression model on intention to getting the second AstraZeneca COVID-19 vaccine in the three health facilities of Lusaka. Controlling for all other factors, a year increase in age increased the odds of intention to getting the second dose of AstraZeneca COVID-19 vaccine by a factor of 1.05 times (95% CI, 1.02–1.08, P = 0.010). Similarly, males had an increased odds of getting a second dose of vaccine (AOR, 1.21; 95% CI, 0.67–2.19; P = 0.399) compared to females. However, this increase was not statistically significant (see Table 3).

**Table 3:** Multivariable mixed effect logistic regression model (combined and stratified analysis).

Variables	Combine	Female	Male
	AOR (95% CI)	AOR (95% CI)	AOR (95% CI)
**Age**	1.05 (1.02–1.08)***	1.05 (1.01–1.10)***	1.03 (0.98–1.09)
**Sex**			
Female	Ref	Na	Na
Male	1.23 (0.68–2.21)	Na	Na
**Marital Status**			
Not Married	Ref	Ref	Ref
Married	0.98 (0.49–1.92)	1.84 (0.75–4.37)	0.35 (0.11–1.08)
**Occupation**			
Unemployed	Ref	Ref	Ref
Unemployed	0.93 (0.47–1.84)	1.16 (0.46–2.90)	1.81 (0.29–2.29)
**Education**			
Upto Primary	Ref	Ref	Ref
Secondary	1.38 (0.28–6.77)	1.31 (0.13–13.15)	1.76 (0.17–17.72)
Tertiary	2.33 (0.51–10.65)	2.40 (0.26–21.89)	3.08 (0.34–27.82)
**Pre-existing Medical condition**			
No	Ref	Ref	Ref
Yes	0.87 (0.39–1.93)	0.65 (0.25–1.70)	1.91 (0.37–9.75)
**Side effects**			
No	Ref	Ref	Ref
Yes	0.59 (0.28–1.84)	0.27 (0.06–1.20)	1.05 (0.42–2.63)
**Took a COVID-19 test before**			
No	Ref	Ref	Ref
Yes	1.69 (0.94–3.02)	1.58 (0.70–3.59)	2.21 (0.87–5.06)

*** = P-value < 0.05 (statistically significant)

Furthermore, those who were married (AOR, 0.98; 95% CI, 0.49–1.94; P = 0.632), developed side effects after getting the first dose of vaccine (AOR, 0.60; 95% CI, 0.29–1.24; P = 0.206) or reported underlining medical conditions (AOR, 0.87; 95% CI, 0.39–1.93; P = 0.632) had reduced odds of getting second dose of the AstraZeneca COVID-19 vaccine. While those who were employed had increased odds of getting the second dose of AstraZeneca vaccine (AOR, 1, 96; 95% CI, 0.53–2.10; P = 0.883) compared to those who were unemployed. However, in the adjusted model only age significantly (P<0.05) predicted intention to getting a second dose in both the combined and stratified analysis (only for females). The selection of the best-fit model involved the use of the AIC and BIC. The likelihood ratio test from the best-fit model also suggested that this model was better than the null model (P = 0.011).

### Margins plot

**Fig 1:**
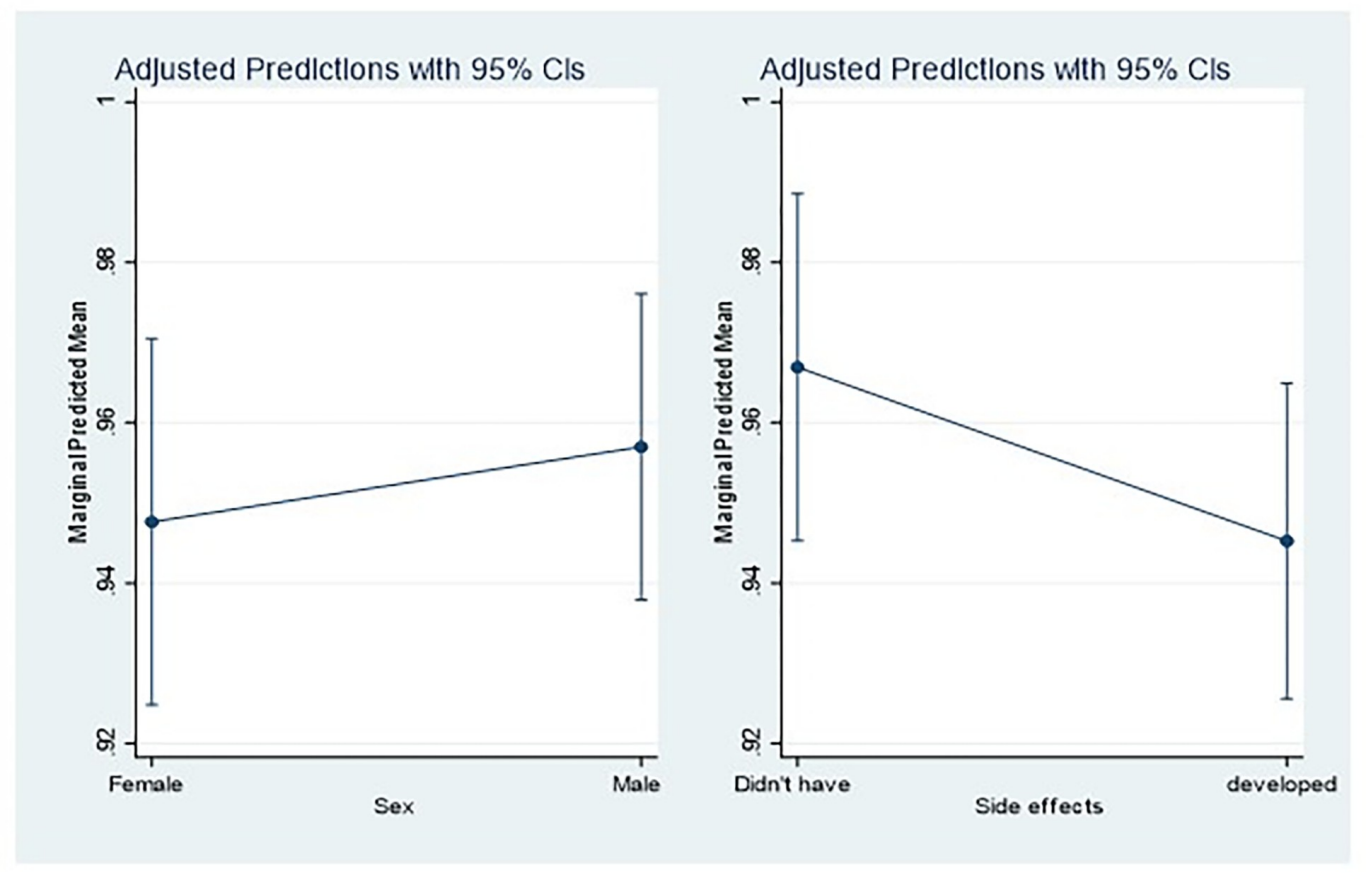
Margins plot on intention to getting a second dose of Astra Zeneca vaccine.

The margins plots were explored and the findings show that males had an increased probability of intention to getting a second dose of the AstraZeneca COVID-19 vaccine compared to females. Similarly, those who never developed side effects had an increased probability of intention to getting the second dose compared to those who developed side effects as shown in Fig 1. The margins probabilities were significantly different from zero (P<0.0001).

## Discussion

This study aimed to evaluate existing gender inequalities, related to intention to uptake of the second dose of COVID-19 AstraZeneca vaccine among an adult population. We have found no significant difference in the proportion of females and males that got the first dose of the vaccine of Astra Zeneca during the period of the study. The median age was not different between males and females. The majority of educated and employed males compared to females got the first dose of the vaccine. The majority of females that got the first dose of Astra Zeneca reported having had pre-existing medical conditions and experiencing side effects after getting the vaccine. Intention to getting the second dose of the vaccine increased with age. Males had an increased probability of intention to getting a second dose of the AstraZeneca COVID-19 vaccine compared to females.

Our finding of no difference in the proportion of females and males that got the first dose of Astra Zeneca is in contrast to findings by Alqudeimat et al (2021) [17] and the Global report for April 2021 [18] that reported male subjects being more likely to accept vaccination against COVID-19 than female subjects. Finding that females and males have an equal likelihood of getting the COVID-19 vaccine suggests lack of gender differences in the COVID-19 vaccine programmes in this context. However, this may not be conclusive as we think a larger study across different regions, rural and urban would provide a wider perspective. This study was conducted in an urban setting where women’s levels of literacy and knowledge about the importance of vaccines may be higher [19]. Further, data for this study was collected during the second biggest COVID-19 which brought in fear and anxiety [20].

Our results provide further significant evidence of female participants reporting post Astra Zeneca COVID-19 vaccination side effects compared to male. This finding is consistent to findings from a cross-sectional study that evaluated the side effects that were experienced by the Bangladeshi residents after receiving the first dose of the Oxford-AstraZeneca’s vaccine which found a significantly higher percentage of female participants suffering from post-vaccination side effects compared to males [21]. Other studies have reported similar findings [22, 23]. One of the reasons for this observation of increased vaccine side effects in females could be due to stronger immune responses in females compared to males [24]. Hence, they are more likely to develop more frequent and more intense side effects. Another reason is the biology of a woman’s body, as estrogen produces a stronger immune reaction against the vaccines [25].

The median age in the study for both females and males who got the vaccine was 40 years. This finding is consistent with other findings that reported that older persons (50 years) got vaccinated [26]. An increase in age also increased the likelihood of the intention to get the second vaccine for Astra Zeneca. In this study, more adults got vaccinated, which could be for fear of having severe infections that could lead to fatality especially that older age and being male are known to be strong drivers of the risk of COVID-19 infection and death [27]. Research shows that individuals who perceive the risk of contracting a vaccine-preventable disease as low, consider the symptoms of the disease as mild, and worry little about the disease, report less intent to take the vaccines, and more often remain unvaccinated [28].

In this study majority of participants, both males and females had acquired higher education level. Existing evidence show that education is a significant factor in people’s views about vaccine safety and efficacy [29], however, there was no statistically significant evidence of an association in our study. Similarly, vaccination in India was greater among the more educated people compared to those who were less educated, yet the findings were not statistically significant [30].

### Limitations in the study

Our results should be interpreted in light of the following limitations. First, this was a cross a sectional study conducted in an urban setting, the findings may not be generalizable to the rural communities to understand the real gender gaps. Second, the study was conducted during the second wave of COVID pandemic with the Delta variant just coming in, therefore, there is a potential that responses to intention to getting the second vaccine may have been influenced by fear of the new variant. Third, we had about 34.7% non-response from our phone call interviews, thus affecting the sample size and potentially reducing the power of the study. Nevertheless, this study remains critical as it reports gender disparities in getting Astra Zeneca vaccine. Data for this study were collected within a short period after participants got their first dose of Astra-Zeneca vaccine, thus minimizing recall bias.

## Conclusion

Findings from our study show that majority of females that got the first dose of AstraZeneca reported having had pre-existing medical conditions and experiencing side effects after getting the vaccine. Finding that the median age of uptake of the AstraZeneca vaccine was 40 years with intention to get the vaccine increasing with age suggests a need for enhancing vaccination programmes targeting young people. The important gender-dependent differences in the side effects reported by females that received the first dose of Astra Zeneca suggests a need for further research to identify specific adverse effects of COVID-19 Astra Zeneca vaccines and close the gender gap in the ongoing COVID-19 vaccination programmes. Further prospective studies with a larger sample size, across regions and with long-duration follow-up, are needed to better understand the side effects in COVID-19 vaccine recipients in the Zambian context.

## Supporting information

S1 Data (DTA)

## References

[1] Du Toit A. 2020. Outbreak of a novel coronavirus. *Nature Reviews Microbiology*, 18, 123–123. 31988490 10.1038/s41579-020-0332-0 PMC7073251

[2] Yassin N., & Saleh S. (2021). The World after COVID-19: Reflections on Global Health and Policy. *Annals of global health*, 87(1), 72. 10.5334/aogh.2902 34327119 PMC8300582

[3] https://www.thelancet.com/journals/lanplh/article/PIIS2542-5196(20)30168-6/fulltext

[4] Koirala A., Joo Y. J., Khatami A., Chiu C. & Britton P. N. 2020. Vaccines for COVID-19: The current state of play. Paediatric respiratory reviews, 35, 43–49. 32653463 10.1016/j.prrv.2020.06.010 PMC7301825

[5] Fontanet A., Cauchemez S. COVID-19 herd immunity: where are we?. *Nat Rev Immunol* 20, 583–584 (2020). 10.1038/s41577-020-00451-5 32908300 PMC7480627

[6] https://zambia.un.org/en/124797-zambia-receives-first-consignment-COVID-19-vaccines.

[7] Knoll MD, Wonodi C. Oxford–AstraZeneca COVID-19 vaccine efficacy. The Lancet. 2021 Jan 9;397(10269):72–4. 10.1016/S0140-6736(20)32623-4 PMC7832220 33306990

[8] Kiely M, Boulianne N, Talbot D, Ouakki M, Guay M, Landry M, et al. Impact of vaccine delays at the 2, 4, 6 and 12 month visits on incomplete vaccination status by 24 months of age in Quebec, Canada. BMC public health. 2018 Dec;18(1):1–5. 10.1186/s12889-018-6235-6. PMC6288945 30537969

[9] Schaffer DeRoo S, Pudalov NJ, Fu LY. Planning for a COVID-19 Vaccination Program. *JAMA*. 2020;323(24):2458–2459. doi: 10.1001/jama.2020.8711 32421155

[10] Macdonald N. E. 2015. Vaccine hesitancy: Definition, scope and determinants. *Vaccine*, 33, 4161–4164. 25896383 10.1016/j.vaccine.2015.04.036

[11] Alleaume C., Verger P., Dib F., Ward J. K., Launay O., & Peretti-Watel P. (2021). Intention to get vaccinated against COVID-19 among the general population in France: Associated factors and gender disparities. *Human vaccines & immunotherapeutics*, 1–12. 10.1080/21645515.2021.1893069 PMC8437523 34292140

[12] Sallam M. COVID-19 Vaccine Hesitancy Worldwide: A Concise Systematic Review of Vaccine Acceptance Rates. *Vaccines* 2021, 9, 160. 33669441 10.3390/vaccines9020160 PMC7920465

[13] https://www.careevaluations.org/wp-content/uploads/Gender-gaps-in-vaccines-October-2021.pdf.

[14] Mathew N, Deborah I, Karonga T, Rumbidzai C. The impact of COVID-19 lockdown in a developing country: narratives of self-employed women in Ndola, Zambia. Health Care Women Int. 2020 Nov-Dec;41(11–12):1370–1383. doi: 10.1080/07399332.2020.1823983 Epub 2020 Oct 8. . 33030978

[15] Carcelen AC, Prosperi C, Mutembo S, Chongwe G, Mwansa FD, Ndubani P, et al. COVID-19 vaccine hesitancy in Zambia: A glimpse at the possible challenges ahead for COVID-19 vaccination rollout in sub-Saharan Africa. Human vaccines & immunotherapeutics. 2022 Jan 31;18(1):1–6. 10.1080/21645515.2021.1948784 PMC8920139 34227914

[16] https://blogs.worldbank.org/africacan/what-driving-COVID-19-vaccine-hesitancy-sub-saharan-africa.

[17] Alqudeimat Y, Alenezi D, AlHajri B, Alfouzan H, Almokhaizeem Z, Altamimi S, et al. Acceptance of a COVID-19 vaccine and its related determinants among the general adult population in Kuwait. Medical Principles and Practice. 2021;30(3):262–71. 33486492 10.1159/000514636 PMC8089409

[18] Global Health 50/50 (2021) The COVID-19 Sex Disaggregated Data Tracker April Update Report.

[19] Parashar S. Moving beyond the mother-child dyad: women’s education, child immunization, and the importance of context in rural India. Social science & medicine. 2005 Sep 1;61(5):989–1000. 15955401 10.1016/j.socscimed.2004.12.023

[20] ALI A, Saeed A, Babar Q. Increasing Fear Of Delta Variant Of New Coronavirus In Pakistan: Strict Actions Required To Wrestle The COVID-19 Peaks. Updates in Emergency Medicine. 2021 Nov 23;1(1):52–3.

[21] Jahan N, Rahman FI, Saha P, Ether SA, Roknuzzaman AS, Sarker R, et al. Side effects following administration of the first dose of Oxford-AstraZeneca’s Covishield vaccine in Bangladesh: A cross-sectional study. Infectious Disease Reports. 2021 Oct 11;13(4):888–901. 34698203 10.3390/idr13040080 PMC8544399

[22] Riad A., Pokorná A., Attia S., Klugarová J., Koščíkm M. Klugar Prevalence of COVID-19 vaccine side effects among healthcare workers in the Czech Republic. J Clin Med, 10 (7) (2021), p. 1428. 33916020 10.3390/jcm10071428 PMC8037149

[23] Menni C., Klaser K., May A., Polidori L., Capdevila J., Louca P., et al. Vaccine side-effects and SARS-CoV-2 infection after vaccination in users of the COVID Symptom Study app in the UK: a prospective observational study Lancet Infect Dis (2021), pp. 939. 10.1016/S1473-3099(21)00224-3 PMC8078878 33930320

[24] Voysey M, Clemens SA, Madhi SA, Weckx LY, Folegatti PM, Aley PK, et al. Safety and efficacy of the ChAdOx1 nCoV-19 vaccine (AZD1222) against SARS-CoV-2: an interim analysis of four randomised controlled trials in Brazil, South Africa, and the UK. The Lancet. 2021 Jan 9;397(10269):99–111. 10.1016/S0140-6736(20)32661-1 PMC7723445 33306989

[25] Acheampong DO, Barffour IK, Boye A, Aninagyei E, Ocansey S, Morna MT. Male predisposition to severe COVID-19: Review of evidence and potential therapeutic prospects. Biomedicine & Pharmacotherapy. 2020 Nov 1;131:110748. 33152916 10.1016/j.biopha.2020.110748 PMC7480230

[26] Painter EM, Ussery EN, Patel A, Hughes MM, Zell ER, Moulia DL, et al. Demographic characteristics of persons vaccinated during the first month of the COVID-19 vaccination program—United States, December 14, 2020–January 14, 2021. Morbidity and mortality weekly report. 2021 Feb 5;70(5):174. 33539333 10.15585/mmwr.mm7005e1 PMC7861480

[27] Robertson E, Reeve KS, Niedzwiedz CL, Moore J, Blake M, Green M, et al. Predictors of COVID-19 vaccine hesitancy in the UK household longitudinal study. Brain, behavior, and immunity. 2021 May 1;94:41–50. 33713824 10.1016/j.bbi.2021.03.008 PMC7946541

[28] Karlsson LC, Soveri A, Lewandowsky S, Karlsson L, Karlsson H, Nolvi S, et al. Fearing the disease or the vaccine: The case of COVID-19. Personality and individual differences. 2021 Apr 1;172:110590. 33518869 10.1016/j.paid.2020.110590 PMC7832025

[29] Solís Arce JS, Warren SS, Meriggi NF, Scacco A, McMurry N, Voors M, et al. COVID-19 vaccine acceptance and hesitancy in low-and middle-income countries. Nature medicine. 2021 Aug;27(8):1385–94. 10.1038/s41591-021-01454-y PMC8363502 34272499

[30] Jain R, Chopra A, Falézan C, Patel M, Dupas P. COVID-19 related immunization disruptions in Rajasthan, India: A retrospective observational study. Vaccine. 2021 Jul 13;39(31):4343–50. 34154863 10.1016/j.vaccine.2021.06.022 PMC8196298

